# Regulation of Hippocampal cGMP Levels as a Candidate to Treat Cognitive Deficits in Huntington’s Disease

**DOI:** 10.1371/journal.pone.0073664

**Published:** 2013-09-05

**Authors:** Ana Saavedra, Albert Giralt, Helena Arumí, Jordi Alberch, Esther Pérez-Navarro

**Affiliations:** 1 Departament de Biologia Cel·lular, Immunologia Neurociències, Facultat de Medicina, Universitat de Barcelona, Barcelona, Spain; 2 Institut d’Investigacions Biomèdiques August Pi i Sunyer (IDIBAPS), Barcelona, Spain; 3 Centro de Investigación Biomédica en Red sobre Enfermedades Neurodegenerativas (CIBERNED), Barcelona, Spain; University of Iowa Carver College of Medicine, United States of America

## Abstract

Huntington’s disease (HD) patients and mouse models show learning and memory impairment associated with hippocampal dysfunction. The neuronal nitric oxide synthase/3',5'-cyclic guanosine monophosphate (nNOS/cGMP) pathway is implicated in synaptic plasticity, and in learning and memory processes. Here, we examined the nNOS/cGMP pathway in the hippocampus of HD mice to determine whether it can be a good therapeutic target for cognitive improvement in HD. We analyzed hippocampal nNOS and phosphodiesterase (PDE) 5 and 9 levels in R6/1 mice, and cGMP levels in the hippocampus of R6/1, R6/2 and Hdh^Q7/Q111^ mice, and of HD patients. We also investigated whether sildenafil, a PDE5 inhibitor, could improve cognitive deficits in R6/1 mice. We found that hippocampal cGMP levels were 3-fold lower in 12-week-old R6/1 mice, when they show deficits in object recognition memory and in passive avoidance learning. Consistent with hippocampal cGMP levels, nNOS levels were down-regulated, while there were no changes in the levels of PDE5 and PDE9 in R6/1 mice. A single intraperitoneal injection of sildenafil (3 mg/Kg) immediately after training increased cGMP levels, and improved memory in R6/1 mice, as assessed by using the novel object recognition and the passive avoidance test. Importantly, cGMP levels were also reduced in R6/2 mouse and human HD hippocampus. Therefore, the regulation of hippocampal cGMP levels can be a suitable treatment for cognitive impairment in HD.

## Introduction

Huntington’s disease (HD) is an autosomal dominant neurodegenerative disorder caused by an expanded CAG repeat in the coding region of the *huntingtin* gene [1]. Although HD is essentially a movement disorder, several evidence indicates that cognitive impairment appears early, even before the onset of motor symptoms, both in patients and mouse models (reviewed in 2). The molecular events involved in this cognitive decline are now beginning to be uncovered. For instance, we recently demonstrated that cognitive dysfunction in R6/1 and R6/2 mice, two exon-1 models of HD, correlates with increased hippocampal cAMP-regulated protein kinase (PKA) activity, and that its inhibition re-establishes recognition memory in mutant mice, supporting the idea that PKA-dependent processes are occluded in HD mice hippocampus [3].

The nitric oxide/soluble guanylyl cyclase/3',5'-cyclic guanosine monophosphate /cGMP-dependent protein kinase (NO/sGC/cGMP/cGK) signaling pathway has been widely implicated in synaptic plasticity, and in learning and memory in different brain regions, including the hippocampus, cerebellum and amygdala (reviewed in 4). NO is produced by nitric oxide synthase (NOS) and stimulates the activity of sGC leading to the production of cGMP [5]. In turn, cGMP can activate cGMP-gated channels [6], modulate the activity of phosphodiesterases (PDEs) [7], and activate cGK, with the consequent phosphorylation of specific proteins involved in signal transduction [8]. Importantly, cognitive loss in Alzheimer’s disease and during aging has been associated with a down-regulation of the NO/cGMP/cGK pathway [9]. However, the integrity of the NOS/cGMP pathway in the hippocampus of HD mice and patients, and the potential contribution of its alteration to learning and memory defects have not been addressed yet. Interestingly, neuronal NOS (nNOS) mRNA levels are decreased in the caudate of HD patients [10], and changes in nNOS protein levels have been also reported in the striatum and cortex of HD mouse models [11–15].

Phosphodiesterases (1–11) play an important role in signal transduction by specifically catalyzing the hydrolysis of the second messengers cAMP and/or cGMP, thereby regulating their intracellular concentration [7]. Evidence from studies in subjects with intact memory and in models of impaired memory indicates that PDE inhibitors can be potentially used as cognitive enhancers [16–21]. Importantly, treatment with sildenafil, a selective inhibitor of the cGMP-specific PDE5 [22], is beneficial in models of cognitive loss associated with aging [23,24] and different pathological conditions including Alzheimer’s disease [25–27], pre-eclampsia [28], and hepatic encephalopathy [29].

The aim of this study was to investigate the nNOS/cGMP pathway in the hippocampus of HD mouse models and patients in order to determine whether it can be a good therapeutic target to improve cognitive function in HD. Our results showed that the nNOS/cGMP pathway is disrupted in the hippocampus of R6 mice and in HD patients and that PDE5 inhibition may prove to be beneficial to ameliorate cognitive deficits in HD.

## Materials and Methods

### HD mouse models

In this study we used male R6/1 and R6/2 heterozygous transgenic mice (B6CBA background) expressing the exon-1 of mutant huntingtin (mhtt) with 145 and 115 CAG repeats, respectively [30,31], and their corresponding wild-type littermates. Male wild-type Hdh^Q7/Q7^ and heterozygous mutant Hdh^Q7/Q111^ knock-in mice were obtained from matings between male and female Hdh^Q7/Q111^ heterozygous as described previously [32]. Mouse genotype and repeat length were determined as described elsewhere [30,33,34]. All mice were housed together in numerical birth order in groups of mixed genotypes, and data were recorded for analysis by microchip mouse number (Avid Identification Systems, Inc., Norco, CA). The animals were housed with access to food and water *ad libitum* in a colony room kept at 19-22^°^C and 40-60% humidity, under a 12: 12 h light/dark cycle. All procedures were performed in compliance with the National Institutes of Health guide for the care and use of laboratory animals, and approved by the local animal care committee of *Universitat de Barcelona* (99/01), and *Generalitat de Catalunya* (99/1094).

### Post-mortem human brain tissue

Hippocampal samples were obtained from the Neurological Tissue Bank of the Biobank-Hospital Clínic-Institut d’Investigacions Biomèdiques August, Pi i Sunyer (IDIBAPS; Barcelona, Spain; URL: www.clinicbiobanc.org), and from the Institute of Neuropathology, Hospital de Bellvitge (University of Barcelona, Spain; URL: www.idibell.cat/modul/biobanc/en), following the guidelines and approval of the local ethics committee (Hospital Clínic of Barcelona’s Clinical Research Ethics Committee). cGMP levels were analyzed in hippocampal samples from six HD patients and five control cases. Details are provided in Table 1.

**Table 1:** Details of control and HD human samples analyzed in the present study.

**Pathological diagnosis**	**CAG repeats**	**Gender**	**Age (years)**	**Post-mortem delay (h)**
None	-	Male	39	3:30
None	-	Male	64	3:30
None	-	Female	71	8:30
None	-	Female	60	15:30
None	-	Female	81	23:30
HD, Vonsattel grade 4	62	Female	28	4:15
HD, Vonsattel grade 4	44	Male	59	5:30
HD, Vonsattel grade 1	40	Male	73	7:00
HD, Vonsattel grade 3-4	n.d.	Male	55	7:00
HD, Vonsattel grade 3	45	Male	53	7:00
HD, Vonsattel grade 3	42	Female	72	17:00

n.d., non-determined

### Determination of hippocampal cGMP levels

Hippocampal cGMP levels were analyzed by using the acetylated version of a commercially available cGMP enzyme immunoassay kit (Sigma-Aldrich, St Louis, MO). The two hippocampi of every mouse were pooled and lysed in 200 μl 0.1 M HCl provided in the kit. Human hippocampal tissue was lysed in 400 μl 0.1 M HCl. Samples were sonicated and centrifuged at 600 x *g* for 15 min at room temperature. The supernatant was collected, acetylated and used according with the manufacturer instructions.

### Total protein extraction

Wild-type and R6/1 mice were killed by cervical dislocation at the age of 8, 12, 20 and 30 weeks, and hippocampi were quickly removed. Tissue was homogenized in lysis buffer [50mM Tris–HCl (pH 7.5), 150 mM NaCl, 10% glycerol, 1% Triton X-100, 100 mM NaF, 5μM ZnCl_2_ and 10 mM EGTA] plus protease inhibitors [phenylmethylsulphonyl fluoride, PMSF (2mM), aprotinin (1μg/ml), leupeptin (1μg/ml) and sodium orthovanadate (1mM)] and centrifuged at 16100x*g* for 20min. The supernatants were collected and the protein concentration was measured using the Dc protein assay kit (Bio-Rad, Hercules, CA).

### Western blot analysis

Western blot analysis was performed as previously described [34]. The primary antibodies used were: anti-nNOS (1:500; BD Transduction Laboratories, San Jose, CA), anti-PDE5A and anti-PDE9A (1:500; Abcam, Cambridge, UK). Loading control was performed by reprobing the membranes with an anti-α-tubulin antibody (1:50000; Sigma-Aldrich) during 20min at room temperature. Then, membranes were washed with TBS-T (Tris-buffered saline containing 0.1% Tween 20), incubated for 1h (20 min for α-tubulin) at room temperature with the corresponding horseradish peroxidase-conjugated secondary antibody (1:2000; Promega, Madison, WI), and washed again with TBS-T. Immunoreactive bands were visualized using the Western Blotting Luminol Reagent (Santa Cruz Biotechnology, Santa Cruz, CA) and quantified by a computer-assisted densitometer (Gel-Pro Analyzer, version 4, Media Cybernetics).

### Sildenafil treatments

To analyze the effect of PDE5 inhibition on cognitive function, 12-week-old wild-type and R6/1 mice received an intraperitoneal (i.p.) injection of vehicle (water) or sildenafil (3 mg/Kg). This dose was selected based on previous studies showing improvement in memory consolidation when sildenafil was administered immediately after training [35–39]. Mice were injected immediately after training in the novel object recognition test (NORT) and in the passive avoidance test, respectively. Memory was assessed 24 h later. The washout period between the two memory tasks was of at least 5 days. Another group of 12-week-old wild-type and R6/1 mice was trained in the passive avoidance test and received an i.p. injection of vehicle or sildenafil (3 mg/Kg) immediately after training. Mice were sacrificed 1 h later, and the hippocampi were quickly dissected, immediately frozen in dry ice, and stored at -80^°^C until analysis of cGMP levels.

### Learning and memory assessment

Learning and memory was analyzed using the NORT [40], and the passive avoidance paradigm [41,42]. Behavioral testing was carried out during the light phase of the animals (ranging from 8: 00 am to 8: 00 pm) in a room maintained in the same environmental conditions as the colony room. The NORT was performed in 12-week-old wild-type and R6/1 mice as previously described [3]. Briefly, mice were first habituated to the arena (circular; 40 cm diameter x 40 cm height) in the absence of objects (3 days, 15 min/day). On the fourth day, a training session was performed during 10 min by presenting two similar objects resembling eggs. Twenty-four hours later, in the testing session, the animals were exposed for 5 min to a familiar and a new object (resembling a cup). The object preference was measured as the time exploring each object (nose spokes) x 100/time exploring both objects. In order to avoid odors, the arena was rigorously cleaned between animal trials by flushing with 70^°^ ethanol and allowing it to dry. The effects of motivation, locomotor activity and anxiogenic components on the learning task were monitored by assessing the distance traveled and the time spent in the center of the open field (automated SMART junior software; Panlab, Spain), as well as the number of defecations. The passive avoidance test is used to assess learning and memory based on the natural preference of mice for a dark environment, and the association between an aversive stimulus (e.g. foot shock) and the preferred environmental context. We used the same mice as for the NORT. The experiment was conducted in a two-compartment device divided by a sliding door (preferred dimly lit compartment; 2-5 Lux; in cm 25 (l) x 25 (b) x 20 (h); brightly lit compartment; 160 Lux; in cm 20 (l) x 15 (b) x 16 (h)). The dark chamber had a stainless steel grid floor for shock delivery. Mice were not previously exposed to the inhibitory avoidance apparatus. On the training day, each mouse was placed into the brightly lit compartment facing to the opposite side of the sliding door. Five seconds later the sliding door was open to allow access to the dimly lit compartment, and the latency to enter the dark compartment (step-through latency) was registered for a maximum of 600 s. Upon entry into the preferred dark compartment with all paws the door was closed and mice received a mild foot shock (1 mA, 2 sec). Twenty seconds later mice were removed from the apparatus and returned to their home cage. Twenty-four hours later mice were returned to the brightly lit compartment, and following a procedure similar to that of training, except that foot shock was omitted (retention test), the latency to enter the shock-paired compartment was recorded for a maximum of 600 s. The retention test was ended when mice stepped completely into the dark compartment, or failed to cross within 600 s. In this case they were assigned a score of 600 s. Retention latency is an index of memory since mice that learn the task avoid the compartment previously paired with the shock, and show greater latency to enter the dark compartment.

### Statistical analysis

All data are expressed as mean ± SEM. Statistical analysis were performed by using the unpaired Student’s t-test (95% confidence) or the two-way ANOVA as appropriate, and indicated in the figure legends. Values of *p*<0.05 were considered as statistically significant.

## Results

### cGMP levels are reduced in the hippocampus of R6/1 mice

**Figure 1:**
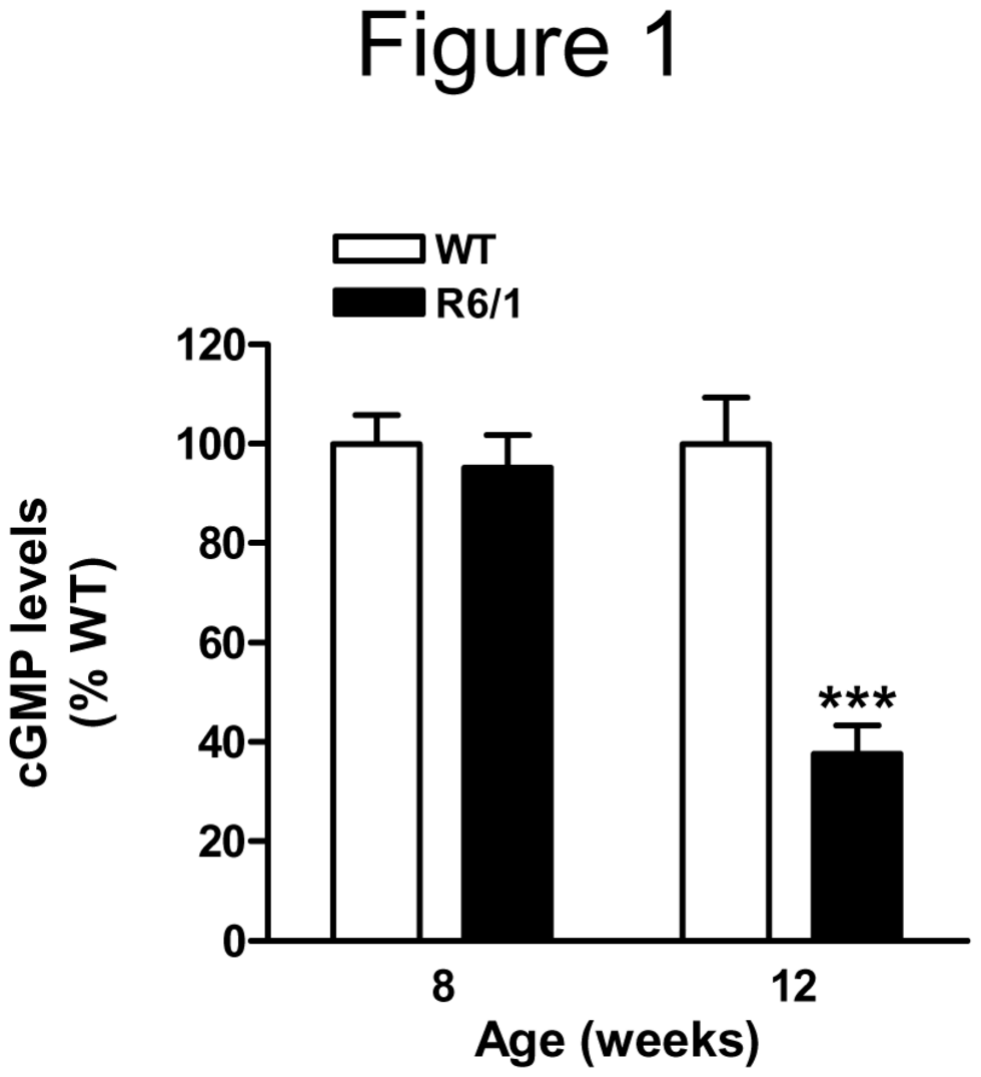
cGMP levels in the hippocampus of R6/1 mice. Hippocampal cGMP levels were analyzed in wild-type (WT) and R6/1 mice at 8 and 12 weeks of age by enzyme immunoassay. Values are expressed as percentage of WT mice, and are the mean ± SEM (n=7-10). Data were analyzed by Student’s t-test. ***p<0.001 as compared with age-matched WT mice.

To address whether changes in cGMP levels might contribute to hippocampal-dependent cognitive deficits in HD mice, we analyzed cGMP levels in the hippocampus of the R6/1 mouse model of HD at 8 and 12 weeks of age. There were no significant changes in cGMP levels in 8-week-old R6/1 compared with wild-type mice (*t*_14_=0.5277, *p*=0.6060; Figure 1). Conversely, at 12 weeks of age, when they show memory impairment [3], cGMP levelswere significantly reduced compared with control littermates (*t*_15_=5.121, *p*<0.001; Figure 1). These results suggest that alterations in the cGMP pathway in the hippocampus of R6/1 mice can participate in cognitive impairment in hippocampal-dependent tasks.

### nNOS levels are down-regulated in the hippocampus of R6/1 mice

**Figure 2:**
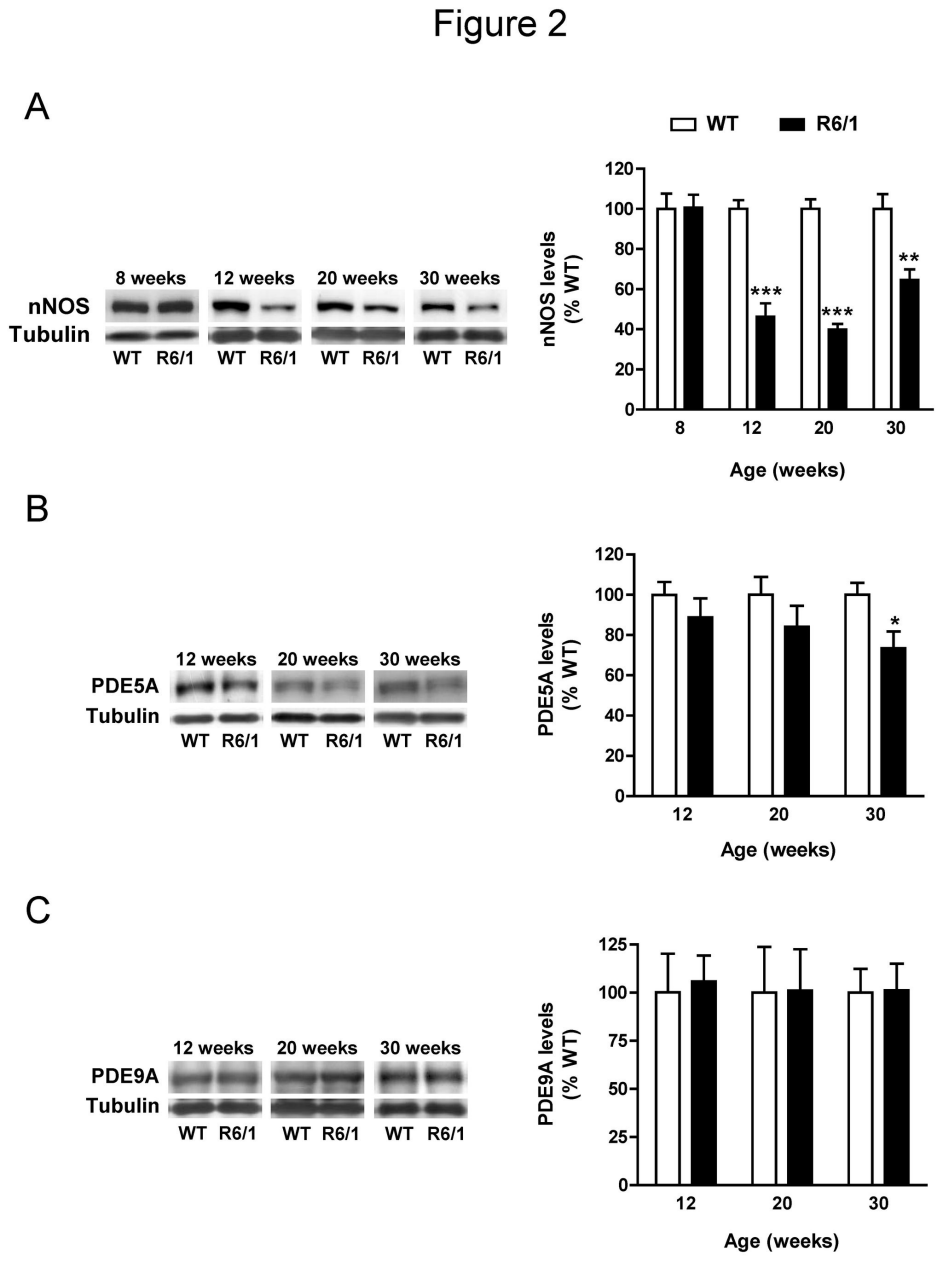
nNOS, PDE5A and PDE9A levels in the hippocampus of R6/1 mice. nNOS (A), PDE5A (B) and PDE9A (C) protein levels were analyzed by Western blot of protein extracts obtained from the hippocampus of 8- to 30-week-old wild-type (WT) and R6/1 mice. Representative immunoblots are shown. Values (obtained by densitometric analysis of Western blot data) are expressed as percentage of WT mice, and shown as mean ± SEM (n=5-8 in A, n=5-12 in B and n=5-7 in C). Data were analyzed by Student’s t-test. *p<0.05, **p<0.01 and ***p<0.001 as compared with age-matched WT mice.

NO activates sGC, which upon activation catalyzes the formation of cGMP. Therefore, the significant decrease in cGMP levels in the hippocampus of R6/1 mice could be related to reduced nNOS levels leading to a lower production of NO. To check this possibility, we analyzed the levels of nNOS in the hippocampus of R6/1 mice at different stages of the disease progression. In agreement with cGMP levels (Figure 1), nNOS protein levels were unchanged in 8-week-old R6/1 mice compared with wild-type animals (*t*_12_=0.064, *p*=0.95), but there was a dramatic reduction in nNOS levels in the hippocampus of 12-, 20- and 30-week-old R6/1 animals compared with age-matched controls (12 weeks: *t*_11_=6.489, *p*<0.0001; 20 weeks: *t*_9_=10.41, *p*<0.0001 and 30 weeks: *t*_11_=3.416, *p*=0.0058; Figure 2A).

In addition to nNOS activity, cGMP levels can also be modulated by PDEs [7]. For this reason, we next analyzed the protein levels of different PDEs, focusing on cGMP-specific PDEs expressed in the hippocampus, PDE5A and PDE9A [43,44]. PDE5A protein levels were unchanged in R6/1 mice at 12 (*t*_11_=0.849, *p*=0.413) and 20 (*t*_14_=1.137, *p*=0.274) weeks of age, and were significantly reduced compared to wild-type animals only at 30 weeks of age (*t*_21_=2.642, *p*=0.015) (Figure 2B). In contrast, PDE9A protein levels were not altered in the hippocampus of R6/1 mice compared with littermate controls at any of the ages analyzed (Figure 2C). These findings indicate that changes in PDE5A and PDE9A levels do not contribute to the reduction of cGMP levels in the hippocampus of 12-week-old R6/1 mice, and that the down-regulation of nNOS is likely the major contributor to reduced hippocampal cGMP levels in the presence of N-terminal exon-1 mhtt.

### Sildenafil treatment increases hippocampal cGMP levels and improves memory in R6/1 mice

**Figure 3:**
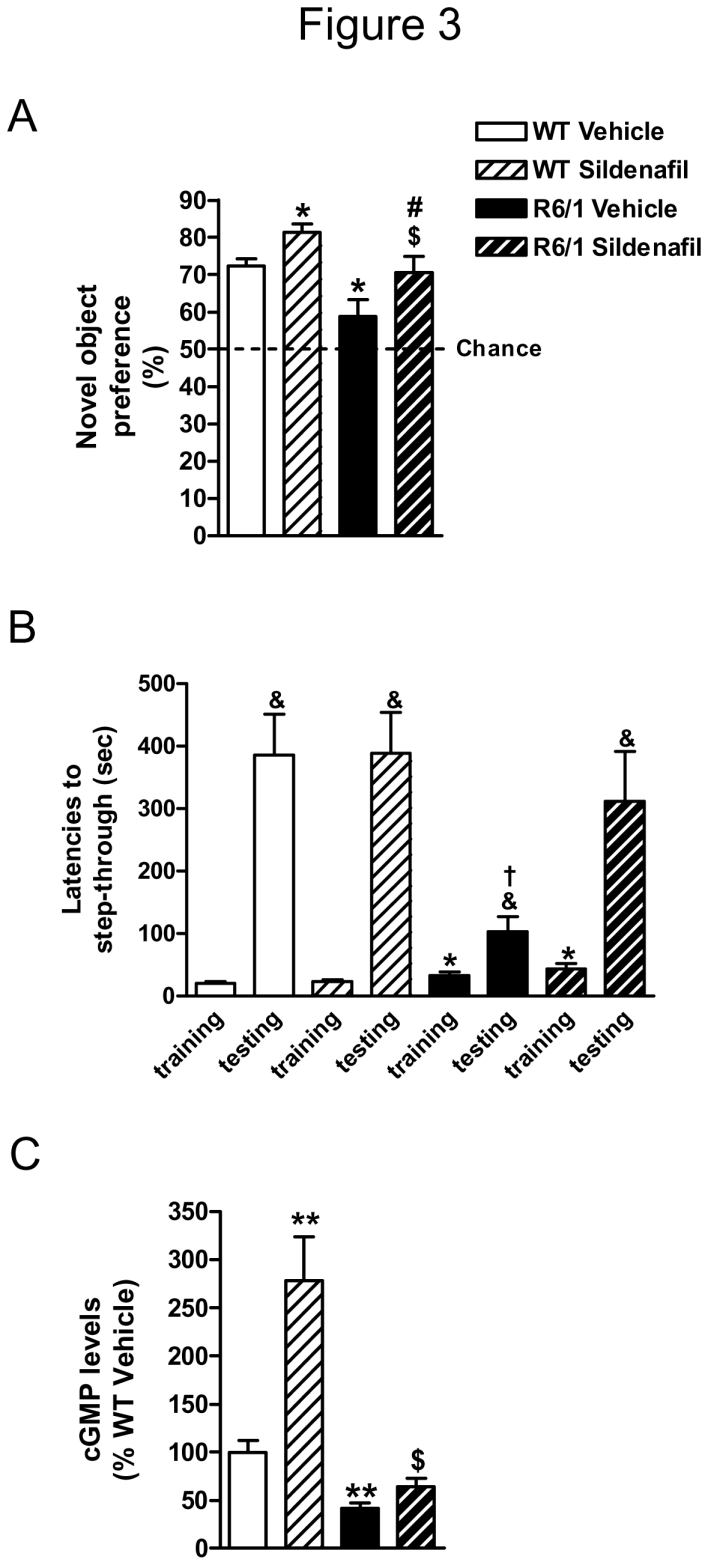
Memory is improved in sildenafil-treated R6/1 mice. Twelve-week-old wild-type (WT) and R6/1 mice received an i.p injection of sildenadil (3 mg/Kg) or vehicle immediately after training for the NORT (A) and the passive avoidance test (B), respectively (n=7-11). Preference for the new object in the NORT (A) and for the brightly lit compartment in the passive avoidance test (B) was quantified 24 h after the training session. A different cohort of mice (n=4-5) was trained for the passive avoidance test, received an i.p. injection of sildenadil (3 mg/Kg) or vehicle immediately after, and was sacrificed 1 h later to determine hippocampal cGMP levels (C). Bars represent the mean ± SEM. Data were analyzed by two-way ANOVA with Bonferroni as a *post hoc* test. *p<0.05 as compared with WT vehicle-treated mice; $*p*<0.05 as compared with R6/1 vehicle-treated mice and #*p*<0.05 as compared with WT sildenafil-treated mice (A). *p<0.05 as compared with latency to step through during training in WT mice groups; & *p*<0.05 as compared with latency to step through during training and † *p*<0.05 as compared with latency to step through during testing in WT vehicle-treated mice (B). **p<0.01 as compared with WT vehicle-treated mice, and $*p*<0.05 as compared with R6/1 vehicle-treated mice (C).

The severe down-regulation of cGMP levels in the hippocampus of R6/1 mice (Figure 1) when cognitive impairment in hippocampal-dependent tasks is evident [3] lead us to hypothesize that pharmacological modulation of cGMP levels could ameliorate cognitive dysfunction in these mice. To address our hypothesis, wild-type and R6/1 mice received an i.p. injection of sildenafil (3 mg/Kg) or vehicle after training in the NORT and passive avoidance test, respectively, and memory was assessed 24 h later. In the NORT, during the training session, there were no differences in the time spent exploring the two objects (data not shown). Conversely, and as previously reported [3], R6/1 mice showed memory deficits (genotype effect: *F*_(1,33)_=14.32, *p*=0.0006) as indicated by their lower preference for the novel object as compared with wild-type mice (Figure 3A). In contrast, sildenafil-treated R6/1 mice showed a higher preference for the new object than vehicle-treated R6/1 animals (treatment effect: *F*_(1,33)_=10.49, *p*=0.0027), and were indistinguishable from wild-type vehicle-treated mice (Figure 3A). Sildenafil treatment also improved memory in wild-type mice (*t*=2.469, *p*<0.05) (Figure 3A). No significant differences were observed in motor activity or anxiety levels during memory assessment (Table 2), indicating that alterations in spontaneous locomotor activity or anxiety levels were unlikely to affect the performance of sildenafil-treated mice in the NORT.

**Table 2:** Anxiety and locomotor activity assessment during the NORT in wild-type and R6/1 mice treated with vehicle or sildenafil.

	**Group**	**Day 1**	**Day 2**	**Day 3**	**Training trial**	**Testing trial**
Defecations	WT vehicle	3.24±0.72	3.10±0.84	2.00±0.22	3.10±1.52	2.32±0.24
	R6/1 vehicle	2.99±2.21	3.40±0.74	3.2±1.33	2.22±0.99	3.11±1.32
	WT sildenafil	3.31±2.10	4.72±0.97	4.2±1.49	3.23±1.08	2.76±1.33
	R6/1 sildenafil	3.00±0.22	3.92±0.41	4.0±0.33	3.69±1.51	3.22±1.40
Time in center (%)	WT vehicle	29.55±2.69	31.17±5.12	31.23±7.55	13.32±4.27	19.31±2.88
	R6/1 vehicle	41.01±7.55	26.41±9.38	33.92±7.66	15.88±5.77	15.65±7.99
	WT sildenafil	32.23±2.22	24.04±6.22	28.55±3.05	14.23±8.11	22.25±6.36
	R6/1 sildenafil	35.75±9.33	25.94±6.81	27.44±6.45	16.55±5.97	25.44±4.54
Distance traveled (cm)	WT vehicle	4525±323	3775±393	3444±252	2223±363	1923±222
	R6/1 vehicle	3800±298	3383±303	2955±422	2099±525	1714±193
	WT sildenafil	4350±234	3815±412	3221±331	3012±495	2013±432
	R6/1 sildenafil	4185±334	3292±368	2978±522	2168±356	1982±297

The number of defecations, time spent in the center of the open field and the distance traveled were analyzed during the NORT in wild-type (WT) and R6/1 mice treated with vehicle or sildenafil (3 mg/Kg) immediately after training. Day 1-3 correspond to habituation. Values are shown as mean ± SEM. For each parameter data were analyzed by two-way ANOVA.

In the passive avoidance test, we found that the step-through latency during the training session was higher in R6/1 than in wild-type animals (genotype effect: *F*_(1,34)_=13.83, *p*=0.0007) (Figure 3B). In the testing session, vehicle-injected R6/1 mice showed a lower latency to step into the compartment previously paired with the shock than wild-type vehicle-treated mice (genotype effect: *F*_(1,33)_=8.791, *p*=0.0056) (Figure 3B). This finding indicated that R6/1 mice suffer memory deficits in avoidance learning. In contrast, sildenafil-treated R6/1 mice showed a recall latency indistinguishable from vehicle-treated wild-type mice (Figure 3B), which further supports the idea that sildenafil treatment improves memory in mutant mice. In this paradigm we did not find improvement in wild-type mice receiving sildenafil treatment after training (*t*=0.032, *p*>0.05) probably because vehicle-treated animals already performed near the threshold (Figure 3B).

Next, we sought to determine whether memory improvement found in sildenafil-treated R6/1 mice was related to higher cGMP levels following training that could contribute to memory consolidation in mutant mice. To this end, wild-type and R6/1 mice were trained in the passive avoidance test, received an i.p. injection of vehicle or sildenafil (3 mg/Kg) immediately after training, and were sacrificed 1 h later to determine hippocampal cGMP levels. We found that cGMP levels were lower in vehicle-treated R6/1 mice compared with vehicle-treated wild-type animals, and that treatment with sildenafil after training increased cGMP levels in both genotypes (Figure 3C). Summarizing, this set of results indicates that increasing hippocampal cGMP levels rescues memory deficiencies in R6/1 mice, and that this can be a good therapeutic target to fight cognitive decline in HD.

### cGMP levels are also reduced in the hippocampus of R6/2 mice and HD patients

**Figure 4:**
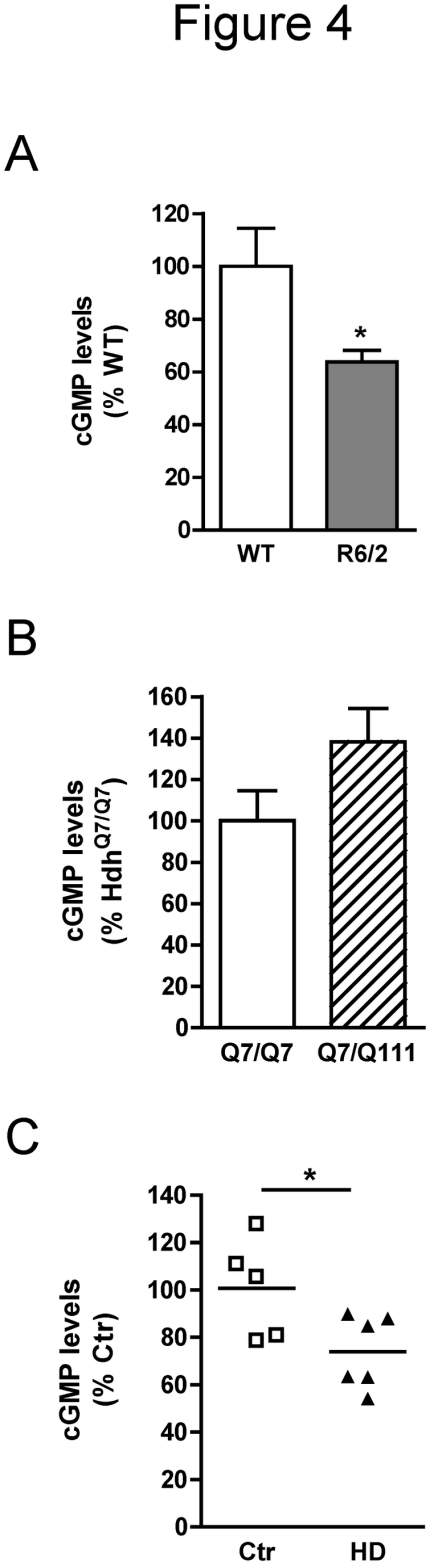
cGMP levels in the hippocampus of HD mice and patients. Hippocampal cGMP levels were analyzed by enzyme immunoassay of samples obtained from 9- to 11-week-old wild-type and R6/2 mice (A), 8-month-old Hdh^Q7/Q7^ and Hdh^Q7/Q111^ mice (B), and control subjects and HD patients (C). Values are expressed as percentage of control mice (A and B; n=6-8) and subjects (C; n=5-6), respectively, and are the mean ± SEM. Data were analyzed by Student’s t-test. *p<0.05 as compared with the control.

We next analyzed if the reduction of cGMP levels found in R6/1 mice hippocampus could be replicated in other HD mouse models when they show cognitive deficits. For this we examined R6/2 mice, which also express N-terminal exon-1 mhtt but show earlier onset and more severe phenotype than R6/1 mice [33], and Hdh^Q7/Q111^ mice, which show late onset and slow progression of the disease [31]. As observed in R6/1 mice, we found that at 9-11 weeks, when R6/2 mice suffer from memory impairment [3], hippocampal cGMP levels were significantly reduced compared with age-matched controls (*t*_13_=2.538, *p*=0.0247) (Figure 4A). In contrast, we did not observe significant changes in cGMP levels in the hippocampus of 8-month-old Hdh^Q7/Q111^ mice compared with age-matched controls (*t*_11_=1.732, *p*=0.1112) (Figure 4B). Finally, we quantified cGMP levels in hippocampal samples from control subjects and HD patients. We found that cGMP levels were significantly reduced in postmortem hippocampal samples from HD patients compared with control subjects (*t*_9_=2.452, *p*=0.0366) (Figure 4C). These results indicate that reduced cGMP levels can contribute to cognitive impairment and that targeting this pathway might also lead to cognitive improvement in HD patients, while cognitive deficits in 8-month-old Hdh^Q7/Q111^ mice [45] are unlikely to be related to alterations in hippocampal cGMP levels.

## Discussion

In this work we show for the first time that the nNOS/cGMP pathway is severely down-regulated in the hippocampus of R6/1 mice, and that PDE5 inhibition improves memory deficits in these animals. Importantly, we also detected decreased levels of cGMP in the hippocampus of R6/2 mice and HD patients, leading us to propose PDE5 inhibition as a therapy to fight cognitive decline in HD.

We detected reduced cGMP levels in the hippocampus of R6/1 mice at 12, but not at 8, weeks of age. Changes in cGMP levels could result from altered synthesis by sGC or increased degradation by PDEs, but we did not detect changes in the protein levels of the cGMP-specific PDE5A and PDE9A in the hippocampus of R6/1 mice at this age. Since we found that, in correlation with cGMP levels, nNOS levels were reduced in 12-, but not in 8-week-old R6/1 mice hippocampus, the alterations in cGMP levels are likely related to diminished nNOS levels, lower NO production and consequent reduced cGMP synthesis by sGC. The finding that nNOS levels were significantly reduced in the hippocampus of R6/1 mice indicates that the levels of this protein are sensitive to the presence of mhtt in several brain regions as nNOS protein levels/NOS activity are also down-regulated in the striatum and cortex of R6 mice [11,14].

We and others have reported that R6/1, R6/2 and Hdh^Q7/Q111^ HD mouse models show object recognition impairment before motor symptoms develop [3,45,46]. Moreover, R6/2 mice display impaired performance in the passive avoidance test [47]. Here, we show that object recognition memory and passive avoidance learning deficits correlate with reduced hippocampal cGMP levels in R6 mice. Accumulating evidence indicates that the activation of the NO/sGC/cGMP/cGK signaling cascade is important during the early phase of memory consolidation. For example, infusion of the cGMP analog 8-Br-cGMP just after the first trial improves memory performance in the object recognition test [48], and in the inhibitory avoidance test [49], while inhibition of nNOS, sGC or cGK impairs object recognition [50]. In addition, hippocampal NOS activity [51], cGMP levels [49,52], and cGK activity [52] increase immediately after training for inhibitory avoidance learning. Thus, the fact that reduced cGMP levels in R6 mice correlate with cognitive deficits suggests an involvement of this pathway in this phenomenon. In contrast, since we did not detect differences in hippocampal cGMP levels between 8-month-old Hdh^Q7/Q111^ and wild-type mice, alterations in cGMP signaling are likely not participating in memory impairment reported in mutant mice [45]. However, we cannot rule out that this pathway might be affected in older animals. The lack of changes in cGMP levels is consistent with the finding that hippocampal nNOS levels were not altered in 8-month-old Hdh^Q7/Q111^ compared with wild-type mice (data not shown). Cortical and striatal nNOS levels undergo a biphasic dysregulation in exon-1 mouse models, with increased levels/activity at early/middle stages followed by a reduction at later stages of the disease progression [13,14]. Thus, it is likely that the late onset and slow progression of the disease in Hdh^Q7/Q111^ mice compared with exon-1 models contributes to these differences.

Importantly, in the present study we showed that memory deficits in object recognition and in the passive avoidance test in R6/1 mice were rescued by the post-training injection of sildenafil. In addition, sildenafil treatment also increased object recognition memory in wild-type mice. Likewise, previous studies have shown that administration of sildenafil, or other PDE5 inhibitors, improves object recognition both in normal subjects, and in models of impaired cognition (reviewed in 18), and ameliorates inhibitory avoidance response in mice showing deficits in this task [53,54]. In contrast, sildenafil-induced memory improvement is abolished in animals receiving an intra-hippocampal infusion of a cGK inhibitor [27,37]. The finding that sildenafil increases cGMP levels and improves novel object recognition memory and passive avoidance learning in R6/1 mice further supports the idea that decreased hippocampal cGMP levels contribute to cognitive dysfunction in these mice.

In addition to the hippocampus (present results and [55,56]), it is noteworthy that sildenafil also increases cGMP levels in the cortex [55,57] and striatum [56], and that PDE5 inhibition improves the performance in cognitive tasks involving these brain regions, both in unimpaired subjects and in models of impaired memory (reviewed in 18). Moreover, it increases NOS activity in the striatum and cortex [58], which might be relevant in the context of HD, as nNOS mRNA levels are decreased in the caudate of HD patients [10]. It is also worthy to mention that treatment with sildenafil restores cGMP levels in pathological situations associated with reduced cGMP in the brain [28,29], improves memory in mouse models of Alzheimer’s disease [25–27], and ameliorates age-related cognitive decline [23,24]. Importantly, it has been recently shown in patients with erectile dysfunction that treatment with a PDE5 inhibitor improves cognitive function [59]. The finding that hippocampal cGMP levels are decreased in R6 mice, and in human HD hippocampus, together with previous results indicating that nNOS pathway is also highly affected in the striatum and cortex of HD mice [11–15], suggests that PDE5 inhibition can be a good therapeutic strategy for cognitive improvement in HD.
